# A81 GASTROINTESTINAL (GI) FEATURES OF HYPERMOBILE EHLERS-DANLOS SYNDROME (HEDS) AND HYPERMOBILITY SPECTRUM DISORDER (HSD): A SCOPING REVIEW

**DOI:** 10.1093/jcag/gwad061.081

**Published:** 2024-02-14

**Authors:** N Chang, D Borovsky, Y Yuan, D Armstrong

**Affiliations:** McMaster University, Hamilton, ON, Canada; McMaster University, Hamilton, ON, Canada; McMaster University, Hamilton, ON, Canada; Medicine, McMaster University Faculty of Health Sciences, Hamilton, ON, Canada

## Abstract

**Background:**

Ehlers-Danlos syndromes (EDS) are a varied group of hereditable connective tissue disorders (HCTD) typified by skin, joint and vascular features, along with less well-recognized features of chronic pain, immunological dysfunction and chronic GI symptoms.

The latest EDS classification (2017) lists 13 EDS types and 19 genetic factors but there is no identified genetic basis for hEDS, the most common type and the most-commonly associated with GI symptoms. There is a need for robust data on the prevalence of GI symptoms and diagnoses in hEDS and HSD.

**Aims:**

To investigate the extent of evidence on GI symptom prevalence and methods used to establish GI diagnoses in patients with hEDS and HSD.

**Methods:**

Following the JBI (Joanna Briggs Institute) guide for scoping reviews, a systematic search of Ovid Medline, Embase and Cochrane Library retrieved scoping and systematic reviews, published in English (1974 – Sept 2023), reporting GI symptom prevalence, assessment and complications in EDS or HSD. Abstracts of identified papers were reviewed by NC & DB and discrepancies resolved by YY & DA. As a scoping review, it required no statistical comparisons.

**Results:**

Duplicate screening of 410 abstracts yielded 10 papers that met inclusion criteria: 1 scoping and 9 systematic reviews. Four systematic reviews reported GI surgical issues related to vascular EDS; none was evaluated further. Two papers reported, specifically, on GI symptoms in patients with postural tachycardia syndrome or eating disorders, some of whom had hEDS or HSD and two papers reported on multiple GI and non-GI symptoms associated with fibromyalgia or functional somatic symptoms. The final 2 papers reported on multiple GI and non-GI symptoms in patients with HSD or multiple EDS types, limited in the latter case to papers published after the latest EDS classification (2017). In general, GI symptoms were characterised as IBS-like symptoms (Rome III or Rome IV) or as typical GI symptoms but without using a validated symptom questionnaire.

**Conclusions:**

There are few data on GI symptom prevalence in hEDS or HSD and no standardised methods for identifying or characterizing GI symptoms or function in hEDS. The evaluation of GI function and symptoms in EDS is hampered by the absence of a definitive genetic or diagnostic test for hEDS or HSD and by changes in EDS classification over the last 50 years.

There is a need for targeted systematic reviews of the prevalence of various GI symptoms in hEDS individuals and of relevant diagnostic methods, to guide further research into the causes of GI dysfunction in hEDS or HSD.

**Funding Agencies:**

None

